# High School Coaches Perceptions of Physicians’ Role in the Assessment and Management of Sports-Related Concussive Injury

**DOI:** 10.3389/fneur.2012.00130

**Published:** 2012-10-05

**Authors:** Nolan Williams, Andrew Sas, Jay Madey, Jeff Bodle, Lauren Scovel, Jonathan Edwards

**Affiliations:** ^1^Department of Neurology, Medical University of South CarolinaCharleston, SC, USA

**Keywords:** sports, concussion, athlete, neurology

## Abstract

Sports concussions are an increasingly recognized common type of mild traumatic brain injury (TBI) that affect athletes of all ages. The need for an increased involvement of trained physicians in the diagnosis and treatment of concussion has become more obvious as the pathophysiology and long-term sequelae of sports concussion are better understood. To date, there has been great variability in the athletic community about the recognition of symptoms, diagnosis, management, and physician role in concussion care. An awareness assessment survey administered to 96 high school coaches in a large metropolitan city demonstrated that 37.5% of responders refer their concussed players to an emergency department after the incident, only 39.5% of responders have a physician available to evaluate their players after a concussion, 71.6% of those who had a physician available sent their players to a sports medicine physician, and none of the responders had their player’s concussion evaluated by a neurologist. Interestingly, 71.8% of responders stated that their players returned to the team with “return to play” guidelines from their physician. This survey has highlighted two important areas where the medical community can better serve the athletic community. Because a concussion is a sport-inflicted injury to the nervous system, it is optimally evaluated and managed by a clinician with relevant training in both clinical neuroscience and sports medicine. Furthermore, all physicians who see patients suffering concussion should be educated in the current recommendations from the Consensus Statement on Concussion and provide return to play instructions that outline a graduated return to play, allowing the athlete to return to the field safely.

## Introduction

Concussion is best managed by those with an expertise in clinical neuroscience along with experience in treating athletes (Kutcher, 2010). Currently, there is no set training path specifically for assessment of neurologic injury in athletes, where there are sports medicine-trained docs without neurologic training and neurologists without sports medicine training, both treating concussion. Because of this discrepancy, there is high variability between providers both in the comfort and knowledge of assessment and management of sports concussion. This goes for both untrained neurologists attempting to care for sports injury and sports medicine physicians attempting to thoroughly assess the nervous system (Kutcher, 2010). In order to establish a consistent approach to be followed by practitioners treating sports concussion, the third International Conference on Sports Concussion formulated a position statement, which defines a return to play protocol and gives expert opinion on all elements of the assessment and management of sports-related concussion (McCrory et al., 2009). Unfortunately, a protocol alone does not account for the differences and unique complexity inherent in the assessment and management of each individual concussive injury (Kutcher, 2010).

Concussions often are incorrectly deemed even by some physicians as minor injuries without obvious evidence of damage to the brain, (Genuardi and King, 1995) yet there may be significant short-term and long-term consequences to suboptimal concussion management. The most immediate and catastrophic short-term consequence of returning a player too early after a concussion is second impact syndrome (SIS), which has been reported to be a rare, but deadly consequence of sustaining a second concussion after not fully recovering from the first concussion (Cantu, 1998). In addition, concussion may increase an athlete’s risk of developing depression (Chen et al., 2008) and chronic traumatic encephalopathy (McKee et al., 2009).

Several recent studies have shown significant changes in long-term memory performance. In two studies by Iverson, amateur athletes sustaining more than three concussions over their career developed cognitive deficits while sustaining two or less was not associated with cognitive dysfunction (Iverson et al., 2004; Iverson, 2006). In a study by Moser et al. (2005), it was demonstrated that subtle, but significant prolongations of the neuropsychological sequelae in those youth athletes with a history of two or more previous concussions. Furthermore, in a study by Guskiewicz, players with a history of previous concussions were shown to be more likely to have future concussive injuries than those with no history than those with no history of concussion (Guskiewicz et al., 2003). These studies highlight the need for expert neuropsychological testing.

A recent AAN position statement was released stating that “[a]ny athlete who is suspected to have suffered a concussion should be… evaluated by a physician with training in the evaluation and management of sports concussions” (Wesolowski, 2010). In one study, physicians were utilized in nearly 50% of cases, but neurologists was utilized only 2.1% of the time and a sports medicine physicians only 1.7% of the time (Meehan et al., 2011). In another study, team physicians were indicated to be most often the responsible party for making return to play decisions (Ferrara et al., 2001). In 2006, six professional organizations came together to better define the role and knowledge needed for the “team physician” in regards to concussion management. This group concluded that the team physician must be aware and able to make “on-field” assessments of all neurologic sports injuries (Herring et al., 2006).

Despite these efforts, physicians historically have had a poor track record in following the current recommendations for concussive injury. According to Genuardi et al. only approximately 30% of discharge instructions for a major, tertiary pediatric hospital were based on the current guidelines for return to play for sports concussion (Genuardi and King, 1995). In another study examining Emergency Room (ER) visits for concussion, 69% of patients diagnosed with a concussion had head imaging, but 28% of patients who were discharged were sent home from the ER without specific instructions for return to play or follow-up for further assessment (Meehan and Mannix, 2010).

It has been shown that the ultimate decision for a player returning to play is determined by a team physician, however this must be done within the context of a multidisciplinary team. This team should include comprehensive concussion surveillance along with baseline and post-injury neuropsychological assessment. There is no one more trained for pre- and post-injury neuropsychological assessment than the clinical neuropsychologist. The neuropsychologist has the skill set needed to provide expert post-injury assessment. This assessment should include both a determination of current cognitive functioning along with an assessment of post-injury neurocognitive and psychological state (Echemendia et al., 2009).

In order to better understand the referral patterns to physicians from the athletic community, a survey of high school coaches and athletic trainers in the form of a physician referral assessment for sports concussion was developed. The idea for this assessment was born out of a lack of academic literature on the perceptions of high school coaches as to which physicians would be the appropriate or most accessible choice for an initial evaluation of sports-related concussion.

## Materials and Methods

### Survey development

The first step for the development of this high school coaches’ awareness survey was a determination if there was a pre-existing policy for physician referral for concussive injury utilized by high school coaches and athletic trainers in a large metropolitan city in the southeast United States. This information was obtained through contacting officials within that city’s Department of Athletics and Physical Education. After contacting these individuals, it was determined that the current method of concussion referral was left to the discretion of the high school coaches and athletic trainers in the city. This independence for referral allowed for a true assessment of the high school coaches’ physician referral preferences. Furthermore, because we chose a state with no mandated referral patterns for concussion, this allowed for an observation of the natural history of high school coaches referral patterns. Furthermore, because our sports neurology program is the only one of its kind in the referral base and because we do not require a referral from another physician and will see unfunded patients, we felt like these normally prohibitive issues allowed for a more naturalistic referral pattern. This awareness assessment survey was designed so it could be administered to a large population of high school coaches and athletic trainers. The content of the assessment is felt to be generalizable to other populations outside of the city of interest. The survey contained 20 questions about their position, the number of concussions seen in the past year, referral to physicians, and return to play guidelines. The survey took approximately less than 10 min to complete.

The questions included the following:

(1) Are you familiar with the Graduated Return to Play Protocol? Yes/No.
(2) If you answered yes to the above question, do you utilize the Graduated Return to Play Protocol? Yes/No.
(3) Do you immediately send a player with concussion to the ER? Yes/No.
(4) Do you have a physician available that you use for assessment and management of concussion? Yes/No.
(5) If you answered yes to the above, the physician is typically a: 1-Pediatrician, 2-Family practitioner, 3-Neurologist, 4-Sports medicine doctor, 5-Internist, 6-Orthopedist.
(6) Does the physician (from the above question) utilize the Graduated Return to Play Protocol? Yes/No.
(7) Do you have any comments, concerns.

IRB approval was obtained for this survey. Consent was implied by clicking the survey. Face validity of the survey was determined by our clinical neuropsychologist.

### Implementation of the survey

The project’s attempt was to represent a cross-section of all athletic trainers and high school coaches within the public high schools of a large metropolitan city in the United States. The survey contained questions both on the availability and type of physician referral as well as management for athletes suffering a concussion. The method of answering the survey questions was clear as it consisted of yes/no and multiple-choice questions. The survey was made available to the high school coaches and athletic trainers via email through a link to our survey, which brought the user to the survey as it was made available in REDCap Survey, an online survey program, which is proprietary to Vanderbilt University (Harris et al., 2009). In order to distribute the survey instrument, a major official within the school district emailed the survey to all athletic directors who then forwarded the email to all high school coaches and athletic trainers within their particular school. This email contained detailed instructions both on how to access the survey and how to complete the survey.

In retrospect, we determined that not including neuropsychologists or pilot testing the survey in the sports community were faults of the study. Neuropsychologists were not included initially because neuropsychologists as an entity do not assess and manage concussion independently in the area where the survey was conducted. Although the survey was reviewed by all the authors, piloting the survey with coaches or others in the sports community may have lead to other changes to the survey that were not previously considered. However, it should be noted that there were no direct comments related to the uncertainty of any questions from the responders in the comments section of the survey.

## Results

This high school coach perception assessment survey was disseminated to all high school coaches and athletic trainers within the county of interest and all answers were collected within 1 month of initially sending the survey. The survey participants for this awareness assessment consisted of a sampling of 160 high school coaches and athletic trainers. Of the 160, there were 101 total responses (63%) from 8 schools within the county. Of these responders, 96 (95.1%) of them were high school coaches and 5 (4.9%) were athletic trainers. The limited number of only five athletic trainer responses (4.9% of responses) prevented significant analysis of their answers in this survey, and their answers were not included in the results. Of the high school coaches that responded, 54 high school coached varsity high school sports teams, two high school coached junior varsity (JV) high school sports teams, and 40 high school coached both JV and varsity. Forty seven responders coached men’s sports, 34 coached women’s sports, and 15 coached both men’s and women’s sports. When asked about education on sports-related concussive injuries, 92 of the 96 high school coaches (95.8%) have received formal training on concussion awareness through either a seminar or web based program. Regarding the number of concussions seen in the past year, the mean was 2.5, with some high school coaches reporting that they did not see any and the maximum number seen being 24 in the past year.

When asked questions regarding physician involvement, 36 high school coaches (37.5%) stated they refer players to the ER after a concussion, while 60 high school coaches (62.5%) do not. Furthermore, 38 of the high school coaches (39.5%) stated that they have a physician available for evaluation of a player with a concussion while 58 high school coaches (60.5%) did not have a physician available for a sports-related evaluation. When asked about physicians giving directions on return to play, 69 high school coaches (71.8%) stated that when a player sees a physician, they return to practice with the graduated return to play guidelines while 27 high school coaches (28.1%) stated that the physician did not provide any graduated return to play guidelines. When asked about what type of physician the high school coaches send their players to, a variety of physicians were used. The vast majority of athletes were sent to sports medicine-trained physicians. Of the 38 high school coaches that stated they refer their players to a physician, 35 (92.1%) sent players to a sports medicine physician, 2 (5.2%) sent players to an orthopedist, and 1 (2.7%) responder sent their players to a pediatrician. None of the high school coaches surveyed referred their players to a neurologist for evaluation of their concussion.

## Discussion

Our findings highlight that not all high school coaches polled have immediate access to a referral network of physicians for their players sustaining concussion. With roughly 60% of the high school coaches responding that they have do not have a particular physician for referral, it is clear that further strides must be made by physicians to be involved in the sports community to provide medical access and awareness of the dangers of sports-related concussive injury. This survey highlights the need for further implementation and expansion of the team approach in treating sports-related concussion by coaches, athletic trainers, and physicians. The care of the an athlete after concussion is still segmented in that coaches should had physicians available to assess their players, physicians should provide clear return to play guidelines to the players, coaches, and athletic trainers, and athletic trainers should be able to monitor these injured athletes and have good communications with physicians and coaches.

This concussion referral awareness survey demonstrated that the majority of high school coaches state that they are referring to sports medicine-trained physicians. Sports medicine physicians are a logical choice in that they are well trained in treating all sports-related injuries including concussions. Interestingly, in our survey no one referred their concussed athlete to a neurologist, who are best to care for neurological injury, but generally do not have sports medicine training. This result while surprising to have no referrals, also makes sense in that the sports medicine physician has a historically larger representation for treating sports concussion. As the care for concussion becomes more defined and complex, this finding may highlight a need for further allotment of concussion educational content within sports medicine fellowships along with highlighting the importance of the team approach to providing medical care for any athlete with a concussion.

Our data suggests that nearly 30% of the time, when a concussed athlete goes to a physician for assessment, the high school coach was not provided with return to play instructions which is consistent with past studies (Meehan and Mannix, 2010). This data was disappointing in that all physicians regardless of specialty who are treating athletes with concussion should be trained thoroughly in the current guidelines of return to play management. It would be ideal for all athletes to return to their teams with a return to play guideline for their high school coach and athletic trainer to follow. This is further evidence that training all physicians in proper neurologic assessment and return to play guidelines is crucial to the recovery of athletes after concussion. With the growing interest in concussion care and prevention, this study gives additional evidence demonstrating that all physicians in the sports medicine community who care for athletes must work together and alongside the high school coaches to develop a comprehensive neurologic assessment for management of our concussed athletes.

### Limitations

In retrospect, our survey was limiting in its scope, but does address important topics in the awareness of physician referral and return to play guidelines in the sports community. We feel that this small survey will open the door for further assessment. We feel that certain specific wording may have been confusing to the high school coaches responding, and the survey is less comprehensive than once thought.

## Conflict of Interest Statement

The authors declare that the research was conducted in the absence of any commercial or financial relationships that could be construed as a potential conflict of interest.

## References

[B1] Cantu R. C. (1998). Second-impact syndrome. Clin. Sports Med. 17, 37–44 10.1016/S0278-5919(05)70060-0 9475969

[B2] Chen J. Johnston K. Petrides M. (2008). Neural substrates of symptoms of depression following concussion in male athletes with persisting postconcussion symptoms. Arch. Gen. Psychiatry 65, 81–89 10.1001/archgenpsychiatry.2007.8 18180432

[B3] Echemendia R. J. Herring S. Bailes J. (2009). Who should conduct and interpret the neuropsychological assessment in sports-related concussion? Br. J. Sports Med. 43(Suppl. 1), i32–i35 10.1136/bjsm.2009.058164 19433423

[B4] Ferrara M. McCrea M. Peterson C. (2001). A survey of practice patterns in concussion assessment and management. J. Athl. Train. 36, 145–149 12937455 PMC155525

[B5] Genuardi F. J. King W. D. (1995). Inappropriate discharge instructions for youth athletes hospitalized for concussion. Pediatrics 95, 216–218 7838638

[B6] Guskiewicz K. M. McCrea M. Marshall S. W. Cantu R. C. Randolph C. Barr W. (2003). Cumulative effects associated with recurrent concussion in collegiate football players. JAMA 290, 2549–2555 10.1001/jama.290.19.2549 14625331

[B7] Harris P. A. Taylor R. Thielke R. Payne J. Gonzalez N. Conde J. G. (2009). Research electronic data capture (REDCap) – a metadata-driven methodology and workflow process for providing translational research informatics support. J. Biomed. Inform. 42, 377–381 10.1016/j.jbi.2008.08.010 18929686 PMC2700030

[B8] Herring S. A. Cantu R. C. Guskiewicz K. M. Putukian M. Ben Kibler W. (2006). Concussion (mild traumatic brain injury) and the team physician: a consensus statement. Med. Sci. Sports Exerc. 38, 395–399 10.1249/00005768-200605001-01671 22089299

[B9] Iverson G. L. (2006). No cumulative effects for one or two previous concussions. Br. J. Sports Med. 40, 72–75 10.1136/bjsm.2005.020651 16371496 PMC2491929

[B10] Iverson G. L. Gaetz M. Lovell M. R. Collins M. W. (2004). Cumulative effects of concussion in amateur athletes. Brain Inj. 18, 433–443 10.1080/02699050310001617352 15195792

[B11] Kutcher J. (2010). Legal issues relating to football head injuries, part II. Before the United States House of Representatives Committee on the Judiciary, Washington, 399

[B12] McCrory P. Meeuwisse W. Johnston K. Dvorak J. Aubry M. Molloy M. (2009). Consensus statement on concussion in sport: the 3rd international conference on concussion in sport held in Zurich, November 2008. Br. J. Sports Med. 43(Suppl. 1), i76–i84 10.1136/bjsm.2009.058248 19433429

[B13] McKee A. C. Cantu R. C. Nowinski C. J. Hedley-Whyte E. T. Gavett B. E. Budson A. E. (2009). Chronic traumatic encephalopathy in athletes: progressive tauopathy after repetitive head injury. J. Neuropathol. Exp. Neurol. 68, 709–735 10.1097/NEN.0b013e3181a9d503 19535999 PMC2945234

[B14] Meehan W. P. d’Hemecourt P. Collins C. L. Comstock R. D. (2011). Assessment and management of sport-related concussions in United States high schools. Am. J. Sports Med. 39, 2304–2310 10.1177/0363546511423503 21969181 PMC3359792

[B15] Meehan W. P. Mannix R. (2010). Pediatric concussions in United States emergency departments in the years 2002 to 2006. J. Pediatr. 157, 889–893 10.1016/j.jpeds.2010.06.040 20708747 PMC2988879

[B16] Moser R. S. Schatz P. Jordan B. D. (2005). Prolonged effects of concussion in high school athletes. Neurosurgery 57, 300–306; discussion 300–306. 10.1227/01.NEU.0000166663.98616.E4 16094159

[B17] Wesolowski K. (2010). New AAN position paper on sports concussion emphasizes five critical procedures. Neurol. Today 10, 32–34 10.1097/01.NT.0000388905.42234.ac

